# Vectored Immunotherapeutics for Infectious Diseases: Can rAAVs Be The Game Changers for Fighting Transmissible Pathogens?

**DOI:** 10.3389/fimmu.2021.673699

**Published:** 2021-05-11

**Authors:** Wei Zhan, Manish Muhuri, Phillip W. L. Tai, Guangping Gao

**Affiliations:** ^1^ Horae Gene Therapy Center, University of Massachusetts Medical School, Worcester, MA, United States; ^2^ VIDE Program, University of Massachusetts Medical School, Worcester, MA, United States; ^3^ Department of Microbiology and Physiological Systems, University of Massachusetts Medical School, Worcester, MA, United States; ^4^ Li Weibo Institute for Rare Diseases Research, University of Massachusetts Medical School, Worcester, MA, United States

**Keywords:** adeno-associated virus, vectors, immunotherapy, gene therapy, vaccines

## Abstract

Conventional vaccinations and immunotherapies have encountered major roadblocks in preventing infectious diseases like HIV, influenza, and malaria. These challenges are due to the high genomic variation and immunomodulatory mechanisms inherent to these diseases. Passive transfer of broadly neutralizing antibodies may offer partial protection, but these treatments require repeated dosing. Some recombinant viral vectors, such as those based on lentiviruses and adeno-associated viruses (AAVs), can confer long-term transgene expression in the host after a single dose. Particularly, recombinant (r)AAVs have emerged as favorable vectors, given their high *in vivo* transduction efficiency, proven clinical efficacy, and low immunogenicity profiles. Hence, rAAVs are being explored to deliver recombinant antibodies to confer immunity against infections or to diminish the severity of disease. When used as a vaccination vector for the delivery of antigens, rAAVs enable *de novo* synthesis of foreign proteins with the conformation and topology that resemble those of natural pathogens. However, technical hurdles like pre-existing immunity to the rAAV capsid and production of anti-drug antibodies can reduce the efficacy of rAAV-vectored immunotherapies. This review summarizes rAAV-based prophylactic and therapeutic strategies developed against infectious diseases that are currently being tested in pre-clinical and clinical studies. Technical challenges and potential solutions will also be discussed.

## Introduction

Infectious diseases are among the biggest threats to our society. They range from ancient maladies, such as malaria and influenza, to modern illnesses, such as human immunodeficiency virus (HIV)-1 and the coronavirus disease of 2019 (COVID-19) pandemic. Many strategies have been developed to cure patients with these diseases and to eradicate the related pathogens. Most vaccines today function by introducing either an inactivated form of the pathogen, a live-attenuated strain, or a protein subunit of the pathogen into the body. This exposure stimulates antigen-specific adaptive immune responses and immunological memory, which can protect the host or mitigate the severity of the infection (1). Vaccines have led to the eradication of small pox and have reduced the global incidence of many other diseases (2). However, certain pathogens have been able to evade vaccine-induced sterilizing immunity *via* various mechanisms. RNA viruses, such as HIV, hepatitis C virus (HCV), and influenza exhibit high genetic variation among diverse strains across different geographical locations. Viral polymorphism can persist even within the same infected individual, thus limiting the utility of vaccines that are based on a single strain (3, 4). In addition, retroviruses have rapidly mutating genomes that permit their escape from adaptive immunity (5). Many pathogens actively suppress inflammation and immunological memory by infecting immune cells and inducing T cell exhaustion, further preventing the formation of sterilizing immunity (5, 6). Broadly neutralizing antibodies (bNAbs) can offer protection against multiple strains. Alternatively, blocking the essential primary host cell receptor with monoclonal antibodies (mAbs) can limit infection by multiple strains. For example, ibalizumab, a CD4-targeting mAb, has received United States Food and Drug Administration (FDA) approval for use in multidrug-resistant HIV-1 infections (7). In addition, many mAbs are under development for the targeted treatment of Ebola, Zika, and COVID-19, among others (8). However, these drugs typically require repeated dosing through intravenous injections and have high production costs (9). Therefore, their practical value is limited, especially in underdeveloped areas of the world. Moreover, the processes involved in generating inactivated virus might damage the native conformation of the antigen; while in the case of subunit vaccines, the antigen is often produced using cell lines of non-human origin, which may have distinctive post-translational modification patterns not native to the virus (10, 11). Collectively, these issues may create antigens with altered conformations, resulting in antibodies that are produced which do not neutralize the real pathogen. These low-potency antibodies also run the risk of enhancing virus entry by assisting viral attachment to the host cell in a phenomenon termed antibody-dependent enhancement (ADE). One of the biggest concerns with the recent COVID-19 pandemic, which is caused by the severe acute respiratory syndrome coronavirus 2 (SARS-CoV-2), is the possibility of vaccine-mediated ADE effect, since similar outcomes have been observed with other coronavirus infections (12). To counter this, Pfizer/BioNTech and Moderna have each developed a vectored strategy, in which mRNA encoding the coronavirus spike (S) protein packaged in lipid nanoparticles (LNPs) is delivered to the body to mediate *de novo* synthesis of the S protein, so that the conformation and topology of the antigen best resembles the native protein that is decorated on SARS-CoV-2 (13, 14). This strategy achieved astounding efficacies of 94% or more in phase III trials (ClinicalTrials.gov Identifier NCT04368728 and NCT04470427).

The ability of viral gene transfer systems to deliver functional genes in the host greatly expands the number of strategies that can be used to fight infectious diseases. One such strategy is to use a recombinant virus as a vector to deliver genes encoding therapeutic molecules, such as neutralizing antibodies (NAbs), bNAbs, therapeutic mAbs, and immunoglobulin (Ig)-related derivatives, for direct expression from the host’s tissues, thus negating the necessity of repeated dosing. Viral vectors may also be used to deliver antigen-encoding genes for vaccination against the antigen. Vectors based on adeno-associated viruses (AAVs) are by far the most popular choice for *in vivo* gene delivery, as a result of their relatively low immunogenicity, high safety profile, broad tropism to a range of tissue types, and their propensity to maintain long-term gene expression. This review will cover recombinant (r)AAV-based immunotherapeutic strategies used to combat infectious diseases. An overview of other viral vectors used in vaccines and immunotherapeutics will be introduced, followed by a general biology of AAV. Subsequently, we will discuss technical challenges and potential solutions to rAAV-vector approaches. Finally, the numerous prophylactic and therapeutic strategies that have been developed over the years for various infectious diseases will be highlighted.

### Overview of Viral Vector Gene Delivery Systems

Viral gene delivery systems take advantage of natural viruses’ inherent ability to evade host defense mechanisms and to transfer genetic cargos inside the cell. In general, viral systems offer better delivery efficiencies to the nucleus than non-viral systems, such as LNPs, naked DNAs, or various polymeric complexes. At the same time, viral vectors are more immunogenic, which can be desirable or unwanted, depending on the specific application (15). Many viral vector platforms based on adenovirus, lentivirus, AAV, Sendai virus, poxvirus, measles virus, baculovirus, and herpes simplex virus vectors, to name a few, have been explored for gene delivery, with the first three being the current most common (16–20).

Adenoviral vectors (AdVs) have a packaging capacity of up to 34 kb, mediate rapid gene expression, can potently activate innate immune responses, and can induce strong Th1-polarized adaptive immunity against transgene products (21–23). These features make AdVs attractive for vaccination against infectious disease outbreaks. Human adenovirus type 5 (Ad5), type 26 (Ad26), and chimpanzee adenovirus ChAdOX1 have been explored as delivery vectors for the coronavirus S gene, with the latter achieving 70% efficacy in a phase III trial (NCT04400838) (24–26). The SARS-CoV2-Ad26 vaccine developed by Johnson & Johnson, recently received from the FDA an emergency use authorization approval. At the same time, the strong immunogenicity of AdVs, as well as pre-existing immunity against Ad5 among various human populations, can negatively affect transduction efficacy, transgene longevity, and can cause untoward side effects (27). Ad5 vectors encoding mAbs or Ig derivatives against respiratory syncytial virus (RSV) and H5N1 influenza A virus hemagglutinin (HA) demonstrated short-term (4 to 14 days) protection in mice against these respective viruses (28, 29). Ad5 delivery of mAbs against anthrax (*Bacillus anthracis*) showed initial protection from a bacterial toxin challenge, but was lost within six months (30). Recombinant AdVs that express HIV-1 proteins to elicit vaccination of patients against HIV-1 infection have been explored, but lacked efficacy in human trials (31–33).

Lentiviral vectors (LVs) utilize the capacity of lentiviruses, most notably HIV-1, to mediate semi-random integration of DNA into the host cell genome to enable long-term transgene expression (34, 35). By deleting the genes that are non-essential to vector production or expressing them in *trans*, LVs can accommodate up to 10 kb of sequence and have the potential to transduce both mitotic and postmitotic cells (23, 36). LVs are commonly used for *ex vivo* gene delivery, most prominently in hematopoietic stem cells (HSCs) and T cells, and are an essential production component in the two FDA-approved chimeric antigen receptor (CAR)-T cell therapies (37). Current LVs have limited tropism profiles, which is a bottleneck for *in vivo* gene delivery in preclinical trials (23, 38–41). However, pseudotyping with envelope proteins from other viruses can enhance tissue targeting (42). Other issues for LVs are their potential for genotoxicity and immunogenicity. Integrase-defective LVs may reduce genotoxicity and have been used for episomal delivery with varying degrees of success (43, 44). Alternatively, packaging LVs with nucleases as protein cargos inside the virion might reduce long-term genotoxicity concerns (45, 46).

### Characteristics of AAV and Their Recombinant Vector Counterparts

**Figure 1:**
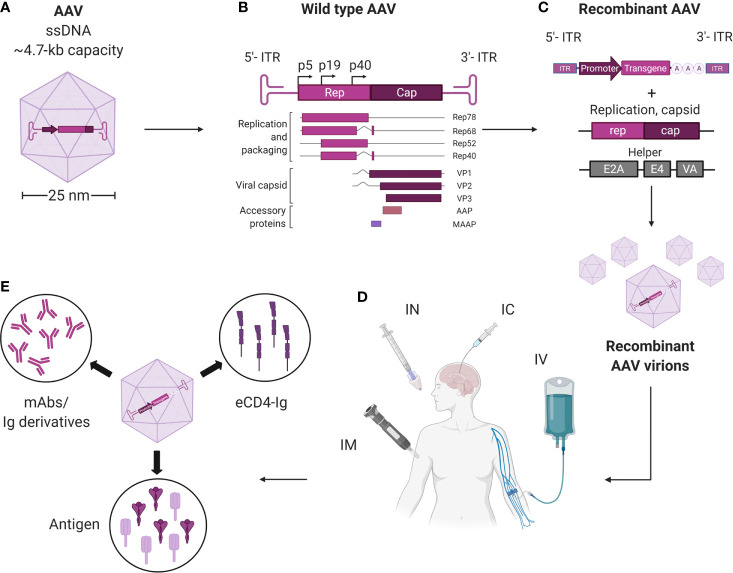
Overview of AAV vectors harnessed for vectored immunoprophylaxis and therapeutics. **(A)** AAVs are small (~25 nm), non-enveloped viruses and have a 4.7-kb, single-stranded, linear DNA (ssDNA) genome encoding four open reading frames. **(B)** *rep* encodes the four genes required for genome replication (Rep78, Rep68, Rep52, and Rep40) and *cap* encodes the structural proteins of the viral capsid (VP1, VP2 and VP3). A third gene, which encodes assembly activating protein (AAP), is embedded within the cap coding sequence in a different reading frame and has been shown to promote virion assembly. The fourth ORF encodes for the recently discovered membrane associated accessory protein (MAAP). The role of MAAP is yet to be clearly defined. **(C)** Providing *rep* and *cap* in trans enables a transgene of interest to be packaged inside the capsid to generate a replication-incompetent vector (recombinant AAV; rAAV). **(D)** rAAVs may be delivered *via* intramuscnular (IM), intranasal (IN), intracerebral (IC), and intravenous (IV) routes. **(E)** rAAVs expressing mAbs, eCD4-Ig and pathogenic antigens can be administered *via* different routes for therapeutics and immunization against infectious diseases. Created with BioRender.com.

Wild-type (wt)AAVs are non-enveloped dependoparvoviruses with a relatively simple architecture (**Figure 1**). Each virion consists of a total of 60 monomers of the capsid proteins (cap), VP1, VP2, and VP3. These proteins respectively assemble at a roughly 1:1:10 ratio into an icosahedral virion that is approximately 25 nm in diameter and 3.9 MDa in size. Multiple AAV serotypes, that differ in cap proteins, are being tested as gene therapy vectors in numerous clinical trials; with each serotype characterized by their unique tissue tropism profiles. Thirteen different AAV serotypes (AAV1-13) and more than 100 natural variants have been reported so far, and more are being discovered. AAV1, AAV2, and AAV9 have been approved for clinical use by the FDA or European Medicines Agency (EMA), and more are being tested in phase I and phase II trials (23, 47, 48). Additionally, the AAV capsid can accommodate various modifications, such as amino acid substitutions, post-translational processing, and chemical alterations (38). This versatility enables the selection of AAV vectors that are better at avoiding pre-existing immunity, targeting the desired tissue, or modulating immune responses.

The encapsidated AAV genome consists of a 4.7-kb linear, single-stranded (ss)DNA that harbors four known open reading frames: *rep*, which encodes for the replication proteins; *cap*, which encodes VP1-3; assembly-activating protein (*AAP*), which promotes capsid assembly within the host cell nucleus; and membrane-associated accessory protein (*MAAP*), whose function is not completely known. The genome is flanked by two T-shaped hairpin structures called inverted terminal repeats (ITRs) (23, 48, 49).

To create rAAV vectors, the genome of wtAAV, save for the ITRs, can be deleted to free up to 4.6 kb of space for transgene cassette packaging (**Figure 1**). Following rAAV transduction, the linear ssDNA genome is converted into circular double-stranded (ds)DNA episomes that reside within the nucleus and are shielded from exonucleases. These episomes are thus highly stable, non-genotoxic, and can be continuously transcribed to enable long-term transgene expression (50–52). For instance, the transgene for the blood coagulation factor IX (F.IX) in one clinical trial patient persisted for more than ten years (53). Alternatively, integration of the rAAV-delivered DNA into the host genome may occur at low frequencies (54, 55). Self-complementary (sc)AAV vectors, in which the sense and the anti-sense sequences of the transgene are packaged inside the vector, can bypass the ssDNA to dsDNA conversion step (23, 48). This allows faster transgene transcription and higher transduction efficiency, but reduces the cargo size by half.

**Figure 2:**
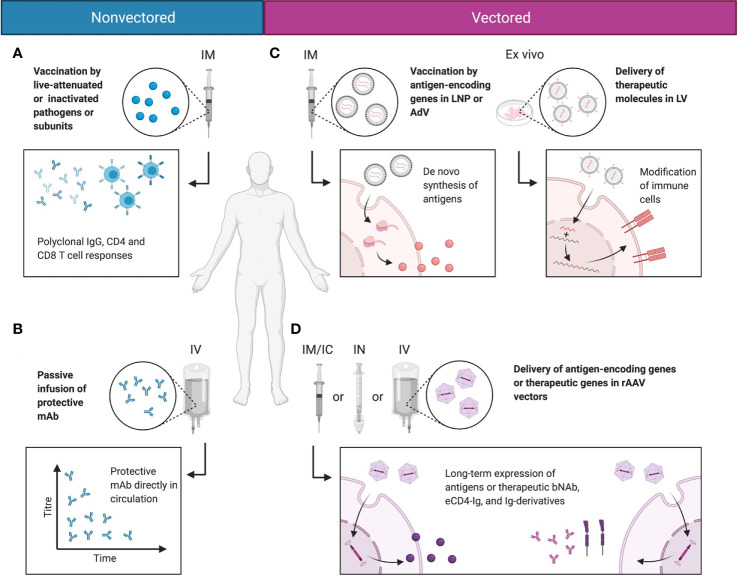
Comparisons between non-vectored and vectored immunotherapeutic strategies. **(A)** Most vaccines today function by delivering antigen in the form of live-attenuated or inactivated pathogens or antigen subunits. This induces polyclonal humoral and cellular responses and immunological memory that protect the host from infections. **(B)** In rapidly mutating pathogens that evade normal vaccine-induced immunity, protective mAbs may be directly delivered to the blood stream *via* passive infusion to mediate protective function. **(C)** Alternatively, genetic material may be delivered in LNPs or viral vectors, such as AdV or LV. This mediates *de novo* synthesis of the antigen in the natural conformation, or allows the modification of immune cells into stronger effector cells. **(D)** rAAV vectors deliver the genetic material of the encoded antigen or therapeutic molecule putatively to the nucleus, as persistent episomes for gene expression. This enables long-term expression of the antigen or therapeutic molecule in a native conformation and topology, and reduces the need for drug redosing. Created with BioRender.com.

On their own, wtAAVs are non-replicating and require helper viruses, such as adenovirus or herpesvirus to complete their life cycle, while rAAV vectors as a single formulation can deliver transgenes to cells both *in vitro* and *in vivo*. In vitro, rAAV is able to transduce many types of primary human and animal cells, as well as cell lines. This ability is highly dependent on the AAV serotype and the cell type (56). Multiple studies have used rAAV vectors to modify cells ex vivo, whereby the cells are isolated from a patient, modified by vectors, and then transplanted back to the host (57, 58). Natural AAV infections are typically asymptomatic in humans. Nonetheless, several recent reports and clinical trials have demonstrated that immune responses were mounted by the host against rAAV vectors and their transgene products. These findings have been covered by multiple comprehensive reviews describing the known immune pathways triggered by rAAV administration (59–61). But overall, rAAV vectors are non-pathogenic with relatively low immunogenicity profiles, making them attractive for *in vivo* transgene delivery. In the context of infectious diseases, rAAV vectors have been used to deliver mAbs and Ig derivatives to achieve prophylactic and therapeutic benefits. Additionally, rAAV vectors have been used as antigenic gene delivery vehicles; because unlike adenoviruses, rAAVs can confer strong immune response against the transgene with minimal response towards the delivery vehicle (62, 63). In certain cases, rAAVs can induce greater and more sustained antibody responses than other vaccination approaches (62). The different modalities for immunotherapeutics, including non-vectored, vectored, and rAAV-vectored approaches, are summarized in **Figure 2**.

### Using rAAVs for *De Novo* Antigen Expression and Induction of Active Immunity

Unlike inactivated, live-attenuated, or subunit vaccines, all of which deliver foreign immunogens in the form of proteinaceous antigens directly to the host, nucleic acids-based vaccines deliver the gene that encodes the antigen for *de novo* synthesis in the host’s cells (1, 2). This strategy simulates the antigen expression process during a natural infection, thus preserving the conformation, topology, multimerization, and glycosylation features of the natural antigen. Conceptually, this should allow the generation of high-quality antibodies that are highly specific to the functional target antigen, and reduce the risk of cross-reactivity and ADE (12). If mutations were to arise during an ongoing infection, the genetic sequence carried by the vaccine vector can be quickly modified without dramatic changes in the manufacturing process; while in the case of inactivated vaccines, the production of variants usually requires many more steps of re-optimization. Despite these advantages, nucleic acids-based vaccines do come with an important concern: the delivery of exogenous antigens into the host may lead to inflammation-mediated toxicity against the transduced tissue. In such cases, vectors can be specifically engineered to target only select tissues without affecting others, *via* cell type-specific expression cassette designs.

Nucleic acids-based vaccine strategies can be broadly categorized into DNA- and RNA-based vaccines. DNA-based vaccines tend to allow longer and more stable antigen expression, but present risks for host genome integration and genotoxicity. In contrast, RNA-based vaccines do not interfere with the genome, but are less stable and may require additional support for optimal function, such as cold-chain storage and booster shots (2, 13, 14). rAAVs are DNA vectors that exhibit relatively low genotoxicity and high stability (64). The first use of an rAAV as a vaccine carrier was in an intramuscular delivery of the herpes simplex virus (HSV)-2 glycoprotein B (gB) or glycoprotein D (gD) antigen to induce active immunity against HSV-2 in mice (65). This treatment resulted in antigen-specific cytotoxic T lymphocyte (CTL) responses and induction of humoral immunity that was more effective than treatments with protein subunits of the antigen or antigen-encoding plasmid DNA. This proof-of-concept study opened a new avenue for rAAV-based genetic vaccines.

### Using rAAVs for Enhancing Humoral Immunity

Strategies for rAAV vectors to deliver therapeutic antibodies or Ig-derivatives to enhance humoral immunity are under development. Humoral immunity is mediated by antibodies, which are highly functional molecules that play critical roles in the host defense against pathogens (66, 67). Each antibody monomer is a Y-shaped molecule with two antigen-binding fragment (Fab) arms and one Fc tail that is capable of host cell ligation *via* Fc receptors (FcR). Upon first encounter with the pathogen, antibodies of the IgM isotype are released from activated B cells. This isotype has a pentameric structure with a total of ten antigen-binding sites that enables high-avidity, low-affinity binding for quick antigen recognition. Activated B cells then undergo clonal selection and somatic hypermutation to release antibodies of the IgG isotype, which exhibit high-affinity binding of the invading pathogen. The vast majority of prophylactic and therapeutic mAbs developed to date are IgG antibodies (68).

Under ideal situations, the above processes eventually lead to the production of potent IgGs that are capable of neutralizing the pathogen, targeting it for processing and degradation in antigen-presenting cells (APCs). In addition, certain IgG-producing B cells gain a memory phenotype that confers long-term immunity against reinfections (69). However, this ideal situation is not always achievable in certain infections, as a result of various pathogen-specific issues. In some cases, the pathogen has highly variable antigens, which may be due to their rapid mutation rates or large geographical distributions. For these pathogens, such as HIV-1 and influenza, rAAV vectors can be used to mediate long-term expression of therapeutic recombinant bNAbs (70, 71). If successful, these approaches would be especially useful for the protection of elderly and immunocompromised people, whose humoral response is impaired.

## Practical Considerations in Developing rAAV-Vectored Immunotherapies

rAAV vectors are showing great potential for treating patients with genetic diseases, but if rAAVs are to be deployed for the prevention and treatment of infectious diseases, additional factors need to be considered. Namely, animal studies of current rAAV-based immunotherapeutics have also revealed many issues to be solved before deployment in a clinical setting. Some of these issues, such as rAAV packaging size and pre-existing immunity against the rAAV capsid, are limitations that are inherent with rAAV-based gene delivery systems. Other challenges, such as anti-drug antibodies (ADAs), mAb-mediated toxicity, and issues related to long-term antigen expression in non-target tissues, are unique challenges for rAAV-based immunotherapy platforms. Advancements in AAV vectorology and virology research may help overcome some of these concerns.

### rAAV Cargo Size

Using rAAVs to deliver mAbs is a well-explored rAAV-mediated immunotherapy modality. A full-length mAb, which is composed of two identical heavy chains (~450 aa) and two identical light chains (~220 aa), requires a ~2-kb packaging space minimum for the cDNA alone. Together with promoter sequences, cis-regulatory elements, and other components, each rAAV vector may only accommodate one full-length mAb (72). This may present a therapeutic challenge, since pathogens like HIV-1 optimally require co-delivery of several bNAbs to better suppress escape mutants (73, 74). Regarding rAAV-vectored bNAb delivery, co-injection of three rAAV1 vectors, each encoding a distinctive bNAb, was explored in rhesus macaques (71). Sustained expression of two out of three bNAbs was observed in one rhesus macaque with low ADA.

If scAAV vectors are used for faster mAb generation, the heavy and light chains of the mAb would need to be packaged into more than one rAAV vector (75). When dealing with cargoes that exceed the packaging capacity of AAV vectors, several strategies can be used (76, 77): 1) once inside the cell nucleus, the vector DNAs may reconstitute into the full-length transgene through homologous recombination; 2) the mRNAs from each cargo can be engineered to undergo trans-splicing into a single mRNA transcript for full-length transgene expression; and 3) the polypeptide products of the cargos can be joined into full-length proteins *via* intein-mediated trans-splicing. The DNA and mRNA recombination-based methods would require co-transduction of the same cell by two rAAV vectors, which can be efficiency-limiting. Intein-mediated protein trans-splicing can presumably work extracellularly and eliminates the need for co-transduction of the same cell (78). Given that mAbs are secreted, intein-based methods may be more appropriate in the context of rAAV-mediated immunotherapy. Alternatively, smaller Ig-derivatives, such as bivalent single-chain variable fragments (scFvs), immunoadhesins, or chimeric Ig-like molecules that combine the functional domain with the Fc domain, may suffice as therapeutic surrogates for full-length mAbs (79–82).

### ADAs

Passively administered mAbs and AAV-delivered mAbs are subject to reduced potencies by ADAs, which come in the form of host antibodies that target exogenous mAbs (83–85). The clinical sequela of ADAs developed against infused mAbs tend be mild. Patients with ADAs may require higher therapeutic mAb doses. But in extremely rare cases, ADA may be associated with anaphylactic shock (85, 86). For AAV-vectored mAb delivery, ADAs are almost universally detected in preclinical studies that looked for them and are considered the main reason for declining therapeutic mAb titers over time. ADAs can appear as early as two weeks post-AAV transduction and animals with higher levels of ADAs usually present with lower levels of therapeutic mAbs in the blood stream (71, 87, 88). On the plus side, no severe adverse events have been documented in preclinical and clinical studies when ADAs against rAAV-delivered mAbs were detected (89).

Developing a generic solution to ADAs is challenging because ADA development is still an enigmatic process. Therapeutic mAbs might be prone to inducing ADAs, as they are large proteinaceous foreign antigens. However, not all hosts receiving the same mAbs develop ADA. This phenomenon is true for passively administered mAbs and rAAV-delivered mAbs in humans (NCT01937455) (71, 87, 89, 90). Also, new mAbs are constantly generated *via* V(D)J recombination and somatic hypermutation during natural immune responses, but no ADAs against endogenous mAbs have been documented. One important difference among endogenous mAbs and rAAV-delivered mAbs is that their site of production is vastly different. The former is endogenously produced in B cells, while the latter is exogenously expressed in other tissues, most commonly the muscle (**Table 1**). Differences in post-translational processing, like the glycosylation of mAbs with non-native cell types might introduce novel epitopes that can potentiate the development of ADAs (125). One study demonstrated that rAAV8-delivered mAbs presented different glycosylation patterns than mAbs derived from infection of cells *in vitro* (88). If these findings hold true in clinical settings, then one possibility of reducing ADAs is to use B cell-targeting rAAV vectors, such as those based on AAV6, for preferential mAb expression *via* gene-edited B cells (126, 127).

**Table 1:** List of AAV-vectored immunotherapeutic strategies.

Pathogen	AAV serotype	Animal	Injection route	Therapeutic mode	References
HIV		Mouse	IM	bNAb	Lewis 2002 (91)
	AAV2/8	Mouse	IM	bNAb	Balazs 2011 (92)
	AAV1	Rh.M.	IM	bNAb-derived immunoadhesins	Johnson 2009 (93)
	AAV1	Rh.M.	IM	bNAb	Fuchs 2015 (70); Martinez-Navio 2019 (71)
	AAV8	Rh.M	IM	bNAb	Welles 2018 (88); Saunders 2015 (94)
	AAV1	Human	IM	bNAb	Priddy 2019 (89)
	AAV1	Rh.M.	IM	eCD4-Ig	Gardner 2015 (95); 2019 (96)
Flu	AAV2/8	Mouse	IM	bNAb	Balazs 2013 (97)
	AAV9	Mouse, Ferret	IN	bNAb	Limberis 2013a (98), 2013b (99); Adam 2014 (100)
	AAV9	Mouse	IN	Multi-domain Ab	Laursen 2018 (101)
	AAV8	Mouse	IM	Nanobody	Del Rosario 2020 (102)
	AAV8	Mouse	IV	2CARD-MAVS	Nistal-Villán 2015 (103)
	AAV9	Mouse	IN	HA/antigen	Demminger 2020 (104)
SARS-CoV-1/2	AAV2	Mouse	IM	RBD/antigen	Du 2006 (63)
	AAV2	Mouse	IN	RBD/antigen	Du 2008 (105)
	AAVrh32.33		IM	Spike/antigen	Vandenberghe and Freeman
	Undisclosed		IN	bNAb	Wilson
Malaria	AAV1, AAV3	Mouse	IM	MSP4/5/antigen	Logan 2007 (106)
	AAV8	Mouse	IM	mAb	Deal 2014 (107)
	AdHu5/AAV1	Mouse	IM	PfCSP/Pfs25/antigen	Yusuf 2019a (108), 2019b (109)
	AAV8	Mouse	IV	miR-155	Hentzschel 2014 (110)
HCV	AAV8	Mouse	IV	NS5B/antigen	Mekonnen 2020 (111)
	AAVrh32.33	Mouse	IM	NS3/4/antigen	Zhu 2015 (112)
	AAV8, AAVrh32.33	Mouse	IM	E2/antigen	Zhu 2019 (113)
HPV/Cervical cancer	AAV1	Mouse	IM	E7/antigen	Zhou 2010 (114)
	AAV5, AAV9	Rh.M.	IN	L1/antigen	Nieto 2012 (115)
Ebola	AAV9	Mouse	IM, IN	mAb	Limberis 2016 (116)
	AAV9	Mouse	IV, IM, IN	mAb	Robert 2017 (117)
	AAV6.2FF	Mouse	IM	mAb	van Lieshout 2018 (118)
Dengue	AAV1	Rh.M.	IM	mAb	Magnani 2017 (119)
	AAVrh32.33, AAV8	Mouse	IM	79E/antigen	Li 2012 (120)
	AAV6, AAV9	Mouse	SC, IM	EDIII/antigen	Slon-Campos 2020 (121)
Prion	AAV2	Mouse	IC	mAb-derived scFv	Wuertzer 2008 (82)
	AAV2	Mouse	IC	mAb-derived scFv	Zuber 2008 (122)
	AAV9	Mouse	IC	mAb-derived scFv	Moda 2012 (123)
Rabies		Mouse	IM	G/antigen	Liu 2020 (124)
					
Anthrax	Ad5/AAVrh.10	Mouse	IV/Intrapleural	mAb	De 2008 (30)
RSV	Ad5/AAVrh.10	Mouse	IV/Intrapleural	mAb	Skaricic 2008 (28)

IM, intramuscular; IN, intranasal; IV, intravenous; SC, subcutaneous; IC, intracerebral; Rh.M., rhesus macaques.

However, several lines of evidence suggests that ADA development, to a large extent, is dependent on the primary sequence of the rAAV-delivered mAb. First, ADA responses are not broad-spectrum, but selective (71). In a triple vector system in which rAAV encoding three different mAbs were delivered to the same non-human primates (NHPs), animals with low ADA response to one mAb can have high ADA response towards the other mAb (71). Second, eCD4-Ig, a modified fusion of CD4 and human IgG Fc, was markedly less immunogenic compared to rhesus-optimized anti-HIV bNAbs (95, 96). ADA responses against eCD4-Ig appeared around four weeks post-transduction and quickly reverted to baseline levels in three out of four NHPs by week 14, while ADA responses against bNAbs persisted until the bNAbs were cleared from the serum. And third, the magnitude of ADA responses in rhesus macaques was positively associated with the degree of sequence divergence from germline V-genes and J-genes (87).

The Fc portion in therapeutic mAbs from non-human origin is thought to drive immunogenicity in human patients (128). However, humanized or fully human mAbs are also immunogenic during passive infusion. Similarly, rAAV-delivered rhesus-optimized anti-HIV bNAbs are immunogenic in rhesus macaques (95, 96). In one study, two murine mAb clones and one human mAb clone were packaged into the same rAAV construct and expressed by the same promoter (116). In recipient mice, the human mAb was expressed at much higher levels than the murine mAbs. While the reason for this observation is unclear, it is possible that the murine mAb-Fc portion might have caused an immune activation against itself, while the human mAb-Fc portion was not as reactive. Alternatively, the Fc portion of an mAB may also potentially lead to complement activation and lysis of the rAAV-transduced cell (93, 129, 130). Nevertheless, there is no experimental evidence that such events happen *in vivo*.

To mediate better bNAb expression, cyclosporin A, an immunosuppressant commonly used during transplantation, was administered to NHPs from 9 days to 28 days after rAAV8-bNAb administration (94). This approach significantly increased average peak bNAb expression from 5 µg/mL to 38 µg/mL, while preserving the capacity of bNAbs to prevent HIV-1 infection. Another method may be to take advantage of liver-induced tolerance of the transgene (131), which primarily results from nonconventional antigen presentation in the liver, causing T cell anergy, apoptosis, and T regulatory (Treg) cell expansion (132). A circumstantial piece of evidence that this strategy may work is that mAb delivery using rAAV8, a serotype with strong tropism in liver, demonstrated less frequent ADA formation than mAb delivery using rAAV1 (~20% ADA-positive in rAAV8 vectors vs. > 50% ADA-positive in rAAV1 vectors) (87, 88). However, there are too many experimental variables between the two studies and direct comparisons are not meaningful.

### Transgene Toxicity

The delivery of antigens in the form of codon-optimized genes *via* a vectored approach will undeniably result in foreign antigen expression in injected or target tissues. Hypothetically, this may lead to inflammation-mediated immunotoxicity against the transduced host tissue. For LNP-vectored mRNAs, this concern is alleviated because mRNAs are inherently unstable and rapidly degraded (2). However, rAAVs are DNA vectors that are capable of mediating long-term antigen expression. To prevent potentially undesirable toxicity, the rAAV vector may be engineered to target specific cell types, such as muscle, without affecting others.

Another major risk is that mAbs are immunologically active molecules with the propensity to activate multiple inflammatory pathways that can mediate tissue damage. When they bind non-specifically, they may cause unanticipated adverse events. Preclinical testing, such as passive administration and human tissue binding studies, can help to avert most of these issues. However, if off-target effects do occur *in vivo*, there is currently no effective method to control antibody transgene expression. As implicated in animal studies, rAAV-mediated mAb expression can persist for life following a single administration of rAAVs (71). In the event that rAAV-mediated mAb is causing off-target toxicity, designing transgene expression cassettes with regulatory elements that can act as on/off switches might solve this problem. Several studies in mice and monkeys demonstrated that rAAV transgene expression levels may be controlled *via* small-molecule drugs (133–135). For example, the mAb may be placed under a rapamycin-regulated promoter (133). Another possibility is to inactivate rAAV-delivered transgenes by destroying the vector DNA. This has been explored in the context of rAAV-mediated CRISPR-Cas9 delivery, in which a second rAAV encoding anti-Cas9 gRNA was able to reduce Cas9 expression (136).

### Pre-Existing Anti-rAAV Immunity

In the context of infectious diseases, successful prevention programs rely on the establishment of herd immunity, whereby a large enough proportion of the population is immunized to cut off the spread of infections. However, pre-existing immunity against AAV capsids significantly limits the pool of eligible recipients (137). Circulating NAbs against AAV serotypes can occur immediately following the disappearance of maternal antibodies (138, 139). Even low titers of pre-existing NAbs can negatively impact vector transduction (140–142). Capsid-specific T cell responses represent another major obstacle for rAAV vector transduction. Prior AAV infections have been proposed to elicit memory T cell responses against the virus (143, 144). These memory CD8^+^ T cell responses are more easily triggered than naïve CD8^+^ T cells and were shown to eliminate host hepatocytes that were successfully transduced by rAAV vectors (141), resulting in reduced or absent transgene expression in clinical trials of rAAV gene therapies for muscle and liver-related genetic diseases (143, 145, 146).

Some direct immunity-inhibiting strategies have been developed to overcome pre-existing immunity against the rAAV capsid, such as using plasmapheresis or IgG-degrading enzymes to remove pre-existing anti-AAV antibodies, or using rapamycin nanoparticles to reduce rAAV immunogenicity (147–149). Unfortunately, these strategies may not be suitable for rAAV-mediated immunotherapy for infectious diseases, because these platforms require an intact immune system. Alternatively, structural modification of NAb recognition sites, directed evolution or rational engineering to generate novel capsids, rAAV epitope masking, chemical modifications, and injecting empty AAV capsids to act as decoys, may be viable evasion strategies against pre-existing immunity. These strategies have been described and reviewed elsewhere (23, 150–163). One potential complication is that, antigen-specific CD8^+^ T cells do not recognize the whole virion but only a short peptide sequence (typically 8 to 14 amino acids in length) presented by major histocompatibility molecule class I (164). Thus, a memory CD8^+^ T cell clone that was induced by one AAV capsid might be cross-reactive against another AAV capsid, if both capsids share the same peptide antigen recognized by the CD8^+^ T clone. Such possibilities, however, have only been demonstrated ex vivo (143). Lastly, administration of rAAV vectors *via* intramuscular, intranasal, or intracerebral routes might be able to reduce or prevent vector encounter with anti-capsid NAbs and T cells (**Table 1**).

### Immune Activation Against rAAV Transgenes

In addition to the anti-AAV NAb and ADA mechanisms discussed above, adaptive immunity against rAAV-vectored immunotherapies can also be encountered in the form of anti-transgene CD4^+^ and CD8^+^ T cell responses, which may result in inflammatory toxicities and other adverse events (165–173). On the other hand, immune activation might be a prerequisite if rAAV vectors are used as a delivery vehicle for vaccination against the delivered antigen.

Innate immunity against rAAVs is remarkably subdued as compared to other viral vectors, but the transgene cargo can be recognized by various pattern-recognition receptors (PRRs) (61). Toll-like receptor (TLR)9 recognizes unmethylated CpG DNA motifs in the genome cargo of rAAV vectors when they are exposed in endosomes and lysosomes, while TLR2 recognizes the AAV capsid. Both of these sensors lead to the activation of type 1 interferons *via* the MyD88 signaling pathway and play vital roles in shaping immune responses (174–182). To reduce TLR9 detection, CpG motifs may be removed from the rAAV cargo (176). More recently, short-noncoding DNA sequences that antagonize TLR9 activation were engineered into the vector genome to prevent the detection of the transgene DNA (183).

To control transgene-specific T cell responses, miRNA-mediated regulation may be exploited to prevent transgene expression from antigen-presenting cells (APCs). For example, binding sites for myeloid-specific miRNA-142 can be engineered into the 3’-untranslated region of the transgene, so that its transcript is destroyed in APCs but not in other cell types (184, 185). Conversely, vectors based on AAVrh.32.33 can induce a robust CD8 T cell response to the transgene product and has been used to express antigens for vaccination purposes (112, 113, 120, 186). Additionally, scAAV vectors induce stronger CD8 T cell responses and humoral responses against the transgene compared to corresponding ssAAV vectors (187).

### Manufacturing and Storage

When deploying rAAV vectors to combat infectious diseases in a large portion of the world’s total population, large production pipelines that can yield consistent quality will be required (188, 189). In addition, current manufacturing processes are prone to introducing empty and partially packaged vectors, thus reducing the purity and efficacy of the rAAV drug, while running the risk of causing deleterious immune activation (190). A standard for rAAV purity should be established, while major innovations in rAAV manufacturing is required. Once rAAV vectors are manufactured, they need to be properly stored to ensure their stabilities. Compared to LNP-mRNA vectors, which currently require stringent storage conditions (-20°C to -80°C) (13, 14), rAAV vectors are generally stable for short periods at room temperature under typical laboratory conditions (64, 186). When distributed as vaccines or mAb carriers, rAAV vectors must confront shipment and handling hazards. Nevertheless, the stability of rAAV vectors might confer an advantage in areas of the world where cold-chain transport facilities are lacking.

## rAAV-Based Immunotherapies in Development

### HIV-1

Despite the advent of antiretroviral therapies, HIV-1 continues to be a major threat to public health, with an estimated 38 million people living with HIV-1 infection and an additional 33 million deceased to date (191). HIV-1 is a lentivirus belonging to the *Retroviridae* family. It harbors two copies of its single-stranded RNA genome, enclosed inside an inner capsid structure and an outer lipid bilayer (192). This lipid bilayer is decorated with HIV envelope glycoproteins gp120/gp41, which use human CD4 as the main receptor, and either CCR5 or CXCR4 as coreceptors for virion attachment and membrane fusion (193). The error-prone nature of reverse transcription, coupled with host-restriction factor APOBEC3-mediated guanosine-to-adenosine mutations, and genomic recombination with other HIV strains, numerous mutations can be introduce into the HIV genome, resulting in antigenic drift, with the potential to evade immune recognition and impart drug resistance (194, 195). No vaccination regimen for HIV has been able to successfully induce bNAb responses. Additionally, HIV specifically targets CD4 T cells and CD4-expressing monocytes, macrophages, dendritic cells, and microglial cells *via* its use of CD4, CCR5, and CXCR4 as receptors/coreceptors. The loss of these essential immune cell populations greatly impairs antiviral response. Finally, HIV can lay dormant inside the host genome without active gene transcription, thus, evading peptide presentation on MHC molecules and detection by T cells (192).

Developing protective humoral immunity against HIV-1 has been particularly challenging. Only bNAbs or antibody clones that recognize multiple strains of HIV-1 have the potential to overcome their high genomic variability (196). Second, each HIV-1 virion has only 4 to 35 glycoprotein Gp120/Gp41 trimer spikes on its surface, while a similarly sized influenza virus has ~450 spikes per virion (197). This means that the monomeric IgG may likely only binds to a single Gp120/Gp41 molecule *via* one of its two Fab arms (197). Thus, the bound IgG is easily detached as a result of its low avidity (197). To stabilize IgG-Gp120/Gp41 binding, the unbound Fab arm must bind to another molecule, usually the virion lipid bilayer or a host transmembrane protein that is incorporated into the envelop during budding and viral egress. This requires the HIV-1-bound IgG to be polyreactive, and in fact, many bNAbs do exhibit a certain degree of polyreactivity and self-reactivity (196). And third, HIV-1 directly impairs CD4 T cells, whose help is critical to proper B cell and antibody development (198). As a result, bNAbs only occur in a minority of HIV-1-infected individuals and only after several years into the infection. Passive infusion using a combination of bNAbs has been able to suppress viremia in HIV-infected patients and offers prophylaxis in simian models (199–201). However, different bNAbs have varied circulatory half-lives and repeated dosing is required, which is impractical. With combinatorial treatments using two bNAbs, the diminishing titer of one bNAb can drive virus evolution towards resistance to the other bNAb (200, 202).

The development of rAAV-based HIV immunotherapies has had mixed, yet promising success. For example, to achieve sustained HIV-1/SIV inhibition in a rhesus macaques model, simian Gp120-specific chimeric immunoadhesins 4L6 and 5L7 (made by fusing select Fabs to the Fc portion of simian IgG2) were packaged into rAAV1 vectors, and tested by IM delivery (93). Four weeks after rAAV transduction, the macaques were challenged intravenously with a lethal dose of SIVmac316. Three out of three with rAAV1-*4L6* and one out of three with rAAV1-*5L7* were completely protected from infection. In another study, macaques were dosed with rAAV1 packaged with *4L6-IgG1* or *5L7-IgG1* transgenes, and then received six intravenous challenges at escalating doses with another SIV strain, SIVmac239 (70). Interestingly, one rAAV1-*5L7-IgG1*-tranduced animal resisted all six challenges, while the other animals presented lower viral loads and slower progression to peak viral loads, indicating that rAAV1-*5L7-IgG1* was partially protective against SIVmac239. This partial efficacy could be explained by the fact that 5L7 conferred strong NK-dependent and antibody-dependent cellular cytotoxicity (ADCC) (70). In another study, rAAV8 vectors carrying anti-SIV mAbs ITS01 and ITS06.02 transgenes were co-administered into macaques by intramuscular injection in a dual vector approach. Animals later received repeated low-dose intrarectal challenge with SIVsmE660 (88). Approximately 90% protection was achieved by these vectors. In a groundbreaking study, a cocktail of three different AAV1 vectors, each encoding a human HIV bNAb (10E8, 3BNC117, or 10-1074), was given to four macaques with preexisting SHIV (SIV-HIV amalgamation) viremia (71). Remarkably, this led to the complete suppression of viremia in a single animal within weeks of rAAV administration. This outcome was maintained throughout the course of the 240-week study, suggesting that a functional cure was achievable. These partial or complete successes in animal models have led to a phase I trial (NCT01937455), in which the HIV-1 bNAb PG9 was packaged into the AAV1 capsid and given to healthy humans at 4 × 10^12^ to 1.2 × 10^14^ vector genomes per subject (89). This approach was well-tolerated, with only mild to moderate side effects that resolved without intervention. However, PG9 could not be detected in the volunteer sera, while anti-PG9 ADA was readily detected in 10 out of 16 volunteers. Only 2 out of 16 volunteers demonstrated neutralization activity against a small number of HIV-1 isolates.

Since CD4 is the universal host receptor for all Gp120/Gp41 variants, one potential solution to counter all HIV-1 strains is to overexpress soluble forms of CD4, which act as decoys to saturate the CD4-binding site of Gp120/Gp41. One such attempt combined parts of CD4 to CCR5mim1, a sulfopeptide that binds CCR5- and CXCR4-tropic viruses. These domains are then fused to the human IgG1 Fc (95). This fusion protein, named eCD4-Ig, or its variants can neutralize a wide panel of HIV isolates, as well as several SIV isolates *in vitro*. The rhesus form of the transgene (*rh-eCD4-Ig*) was delivered intramuscularly by an AAV1 vector (AAV1-*rh-eCD4-Ig*) into macaques. Upon repeated IV challenge with SHIV-AD8, none of the vector-transduced animals became infected, even at the highest dose (95). AAV1-*rh-eCD4-Ig*-transduced macaques were also protected from infection by SIVmac239, of which the gp120/gp41 complex is highly divergent from SHIV-AD8 (96). Escalating doses of SIVmac239 eventually infected all AAV-*rh-eCD4-Ig*-transduced macaques, but the viruses also developed escape mutations that came with fitness costs. Nonetheless, the research for rAAV-based HIV vaccines is ongoing and further advancements are necessary to ensure these therapies are more efficacious.

### Influenza

Influenza infections are the seventh leading cause of death in the United States, with more than 20,000 fatalities recorded in the last year (203). Illnesses range from mild to severe, and even death. Hospitalization and death occur mainly among high risk groups. Individuals with a reduced capacity to mount an immune response upon infection have an increased susceptibility to influenza infections and complications, which include fatal pneumonia and acute respiratory distress syndrome (ARDS) (204). Vaccines are pivotal for influenza prevention, but their efficacies are substantially reduced in the elderly (205–207).

Unlike HIV-1, protective humoral immunity is easily generated against the flu virus by vaccination. However, the flu genome can undergo antigenic shift, a unique influenza virus-associated phenomena that poses additional difficulties (208). The influenza virus is made up of eight segmented negative-sense RNA strands enclosed inside a lipid bilayer that is studded by glycoproteins hemagglutinin (HA) and neuraminidase (NA). When the same organism is infected by multiple influenza subtypes, the RNA segments can be reshuffled to produce novel subtypes in a process called antigenic shift. In addition, influenza RNA polymerase is also highly error-prone, leading to the accumulation of mutations *via* antigenic drift (209).

Most antibodies produced in response to seasonal flu vaccines target the receptor binding site (RBS) within the globular HA-head region (210). Although functional against the vaccinated subtype, they can be rendered less effective by HA mutations and reshuffling. High-affinity bNAbs that can bind a broad array of influenza viruses have been isolated (211–214). They can offer protection by inhibiting fusion of the viral and cellular membranes (215–217), or by Fc receptor (FcR)-mediated mechanisms *via* ADCC (218). However, bNAbs usually target the more conserved regions of HA, which are less accessible than the globular head region and are thus more difficult to vaccinate against (215–217). Variable regions from the heavy and light chains of F10 and CR6261 bNAbs packaged into rAAV2/8 have been tested by administration into mice by intramuscular injection (97, 214, 217, 219). This treatment led to bNAb expression in mice within one week of rAAV administration, and protected mice against lethal influenza challenges with different H1N1 strains (97). Another study made a recombinant antibody-like molecule by fusing alpaca-derived single domain antibody (nanobody) to Fc domains (102). This construct named R1a-B6-Fc was delivered using an rAAV8 vector *via* intramuscular injection, resulting in high-level transgene expression in sera for at least six months, and conferred complete protection against lethal H1N1 and H5N1 challenges.

Given that the respiratory tract is the primary target of influenza virus, the possibility of applying rAAV vectors intranasally has also been explored (104, 220). This administration route is thought to be superior. Intranasal delivery of rAAVs and its transgene product are targeted to nasal epithelia, and has the ability to circumvent pre-existing anti-AAV immune responses, while providing passive immunization. As a proof-of-concept, a potent bNAb against various influenza A subtypes (FI6) (98, 99), was designed into an rAAV9 vector and delivered into mice and ferrets *via* intranasal distillation (98, 100). This afforded protection against several clinical isolates of H5N1 and H1N1, and provided partial protection in mice against clinical isolates of H7N9 (99). An alternative design packaging a humanized, multidomain recombinant antibody (MD3606) into an rAAV9 vector, protected mice against mouse-adapted influenza H1N1, H3N2, and B viruses following intranasal instillation (101). Investigation with NHPs and humans in clinical trials should provide insight into whether these approaches will be efficacious and more effective than conventional vaccines.

### Dengue

Dengue viruses (DENVs) are members of the *Flaviviridae* family and are comprised of four distinct serotypes (DENV1-4) (221, 222). DENVs are enveloped and have a plus-stranded RNA genome. DENV infections may be asymptomatic or characterized as dengue fever (DF), dengue hemorrhagic fever (DHF), and dengue shock syndrome (DSS). There are approximately 3.9 billion people who are at risk of dengue virus infection around the world. Up to 390 million people are infected with dengue virus annually in over 100 endemic countries, with 70% of the actual burden being in Asia (223, 224). DENV is primarily caused by the spread of mosquito vectors and the growth of worldwide travel, and represents a significant global public health problem. Development of safe and effective immunotherapeutics and vaccines are thus a top priority that have yet to be met.

The major challenge for humoral immunity against dengue viruses (DENVs) is avoiding ADE, where the presence of specific antibodies actually enhances DENV pathogenesis (225). The DENV coat is decorated with viral envelope (E) and membrane (M) proteins (221, 222). Without pre-existing DENV antibodies, DENV E proteins bind to specific host cell receptors, leading to virus uncoating and genome release. Infection by one DENV serotype leads to production of antibodies that are cross-reactive with other serotypes. These cross-reactive antibodies do not neutralize DENV, but instead tag the virion for FcR-mediated endocytosis and productive infection (226). Several lines of evidence suggest that these cross-reactive antibodies can increase DENV disease severity. First, mAbs induced by DENVs can increase virus infection of FcR-bearing cells (227). Second, passive immunization of mice with DENV antibodies produced higher viremia following DENV infection, resulting in the death of the infected animals (228). And third, in human populations with high rates of DENV re-infections, the presence of low-titer DENV-specific antibodies is associated with a higher risk of severe disease when patients are infected with a different serotype (229, 230). These outcomes pose significant challenges to DENV vaccine development. As a matter of fact, Dengvaxia, the only licensed DENV vaccine, is only recommended in populations with prior DENV infections, as it is thought to increase dengue disease severity in naïve individuals (231). On the other hand, re-infection with the same DENV serotype leads to efficient neutralization by pre-existing antibodies (232–235).

Given the nature of ADE in DENV immunotherapy, it may be feasible that a cocktail consisting of four AAV-mAb constructs, each encoding a recombinant antibody that can specifically neutralize one of the four DENV serotypes, may protect the host against all serotypes. In addition, the mAbs inside AAV-mAb constructs may be selected *a priori*, for those that lack ADE capacity. This precise strategy has not been attempted. Instead, anti-DENV3 NAb P3D05 was packaged in rAAV1 and delivered to macaques *via* intramuscular injection (119). Despite the high P3D05 expression that lasted for months, DENV3 infection, replication, and the development escape mutants were unaffected. Better understanding of the interactions between NAbs and DENVs, and screening of stronger NAbs are necessary to improve vector design and transduction outcomes.

### SARS-CoV-2

COVID-19 emerged during the fall and winter of 2019 and was declared a global pandemic by the World Health Organization on March 11, 2020 (236, 237). As of February 27^th^, 2021, more than 100 million people worldwide have been infected with SARS-CoV-2 and more than two million deaths have been reported (238). SARS-CoV-2 is a coronavirus composed of a positive-sense RNA genome that is enclosed inside a lipid membrane, which is heavily studded by transmembrane S proteins (239). The S protein contains the receptor binding domain (RBD) and directly interacts with the host angiotensin-converting enzyme 2 (ACE2) receptor, which is essential for virus attachment and cell invasion (239, 240). Current vaccine development has focused on inducing antibody responses against the S protein. However, previous studies on SARS-CoV, a virus with ~80% sequence identity with SARS-CoV2, suggests that ADE induction may be a concern. Some studies have demonstrated that anti-S antibodies, or certain vaccine compositions, could increase virus uptake in macrophages, elevate pulmonary infiltration of proinflammatory cells, and exacerbate lung injury. Other studies have shown that immunization reduces viral load and protects the lung from severe damage (241–244).

After examining the available evidence from SARS-CoV and DENV studies, it appears that the key to avoiding ADE, while achieving antibody-mediated protection, is to generate highly specific NAbs at high doses without inducing low-potency antibodies that bind, but do not neutralize (12). To achieve this outcome, the vaccinating antigen must resemble the native antigen; hence, the majority of COVID-19 vaccine designs use the S-2P recombinant protein that contains two proline insertions to stabilize the vaccinating antigen (240). Two such designs, one by Pfizer/BioNTech and another by Moderna, demonstrated greater than 94% efficacy in phase III trials and are currently being distributed in the United States under emergency use (13, 14). The underlying mechanisms for achieving such high efficacies in these two vaccine compositions are still being investigated, but NAbs against SARS-CoV-2 are thought to play an important role (245–247).

Recently, an rAAV platform called AAVCOVID, which borrows from the same principle of expressing the S protein antigen by packaging the SARS-CoV-2 S gene within an AAV capsid called AAVrh32.33 is being explored as an experimental vaccine (186, 248). The basis of the strategy relies on two previous findings. First, AAVrh32.33, a hybrid of two AAV natural capsid sequences isolated from rhesus macaque, has been shown to induce high antibody titers and potent CD8+ T cell responses in mice and non-human primates (249–251). And second, rAAV2 vector expressing the RBD of SARS-CoV, the virus responsible for the 2002 SARS epidemic, can induced sufficient neutralizing antibody against SARS-CoV infection with a single intramuscular injection (63). In addition, intranasal instillation can induce a strong local humoral response, and elicited stronger systemic and local specific cytotoxic T cell responses than intramuscular injections. Nevertheless, the protection against SARS-CoV challenge was comparable for both modes of administration (105).

Another immunotherapy strategy is to directly deliver a cocktail of neutralizing mAbs (casirivimab and imdevimab) that target different regions of the RBD, in order to prevent further viral spread in patients (252–254). This method was recently granted an emergency use authorization by the FDA in certain high-risk patients with mild to moderate COVID-19 (255), and a phase I-III trial is ongoing to analyze its safety and efficacy (NCT04425629). The interim results suggest that intravenous and subcutaneous administration of the cocktail resulted in mAb presence for a month or more, was generally safe, and reduced viral loads in COVID-19 patients. Based on these preliminary results, research is underway to investigate whether intranasal administration of rAAVs carrying casirivimab and imdevimab can confer long-term mAb expression in the nasal mucosa for prophylaxis against COVID-19 (256).

### Prion Disease

Prion diseases, or transmissible spongiform encephalopathies (TSEs), are caused by the misfolding of the normal cellular prion protein (PrP^C^) into the abnormal pathogenic scrapie isoform (PrP^Sc^), or simply prions (257). Unlike PrP^C^, prions are resistant to protease digestion and are capable of converting normal PrP^C^ into more prions. Prion accumulation primarily affects the central nervous system (CNS) and leads to a series of neurological degenerative disorders that inexorably ends in death. Drug development for prion diseases is challenging, because in order to gain access to prions, the drugs must cross the blood brain barrier to efficiently reach the CNS. It has been demonstrated that anti-PrP^C^ mAbs could protect the normal PrP^C^ from interacting with the pathogenic prion and slow disease progression (258, 259). However, these antibodies and other compounds typically show limited efficacy in long-term animal studies. This failure is likely due to host tolerance to endogenous PrP^C^ and poor antibody diffusion into neuronal tissues (260–264).

The natural tropism of certain AAV serotypes towards the CNS presents a huge advantage for rAAV-vectored mAb delivery (265). To this end, several studies have tested rAAV vectors that deliver scFv proteins *via* the intracerebral route (82, 122, 123, 266). These scFvs were made by fusing the variable regions of the heavy and light chains of an mAb clone, which preserved the antigen-recognition capacity of the original mAb, but lacks Fc-mediated function. Intracerebral delivery of rAAV2 or rAAV9-packaged scFvs that binds to PrP^C^ can delay prion disease onset in mice (82, 123). An rAAV2 vector that delivers scFvs against the laminin receptor, which interacts with both PrP^C^ and prions, also delayed onset of prion pathogenesis and decreased PrP^Sc^ burden in the CNS of mice (82, 122, 123, 266). Further studies are needed to assess the translatability of the platform in humans.

### Filoviruses

Filoviruses, which include Ebola virus, Marburg virus, and others, are notable for their capacity to cause highly lethal infections in humans (267). A live attenuated vaccine, trade-named Ervebo, is available for *Zaire ebolavirus*, a highly pathogenic strain of Ebola (268). This vaccine consists of a pseudotyped vesicular-stomatitis virus (VSV) that expresses *Zaire ebolavirus* glycoprotein (GP) and has near 100% efficacy. However, no vaccines are yet available for other filoviruses, which tend to cause sporadic outbreaks and lack exhaustive study (269). Furthermore, no targeted treatments are available for patients with symptomatic infections.

Several studies have demonstrated that mAbs raised against the surface glycoprotein (GP) of Zaire ebolavirus are capable of protecting experimental animals from lethal Ebola virus infections within a therapeutic window of three days pre-challenge to five days post-challenge (270–273). In light of these findings, several groups examined whether rAAV vectors can be used to deliver long-lasting Ebola-specific mAbs for durable prophylaxis and therapeutics. *Zaire ebolavirus* GP-recognizing mAbs were designed into rAAV9 vectors and delivered into mice *via* intramuscular, intravenous, or intranasal routes. Vector-treated animals showed rescue and longer survival from lethal Ebola challenges than animals subjected to mAb infusion (116, 117). Injection by intravenous and intramuscular routes offered better protection against Ebola virus as compared to intranasal administration, and the level of protection significantly increased with vector dose. Notably, an AAV6 variant, called AAV6.2FF, was used to package Ebola-specific mAbs (118). In this study, treatment with a single vector rAAV6.2FF-mAb clone presented 83% to 100% efficiency, while a dual therapy using two vectored mAb clones resulted in complete protection. The protective effect lasted for at least five months after a single rAAV6.2FF-mAb administration. However, treatment with a seven-day lead time was required for complete protection, suggesting that this method of mAb delivery may be too delayed for treating patients with symptoms, since the entire disease course spans only 14 to 21 days (274).

### Anthrax and RSV

Some pathogens require a rapid response to block progression of the disease. To promote rapid transgene expression, one strategy is to use a dual-vector approach with an initial AdV treatment to mediate rapid expression, followed by an rAAV vector to mediate long-lasting expression. This formulation is explored in treating infections by *B. anthracis*, a potential bioweapon in terrorist attacks (275), and in RSV, a common cause of respiratory disease in infants and adults (276).

Antibodies against anthrax protective antigen (PA) are effective at inhibiting anthrax lethal toxin (LT)-mediated damage (277). To achieve rapid inhibition of toxin, a dual-vector platform comprising of recombinant anti-PA antibody packaged into Ad5 and AAVrh.10 vectors was developed (30). The Ad5-anti-PA and AAVrh.10-anti-PA vectors were then administered into mice *via* intravenous and intrapleural injection routes, respectively. While Ad5-anti-PA conferred complete protection against LT challenge between one day and eight weeks post-injection, the AAVrh.10-anti-PA conferred complete protection against LT challenge from two weeks to 26 weeks. When both vectors were given together, complete protection was observed from day 1 to week 26 post-injection.

The dual-vector strategy for RSV utilizes the murine form of the anti-RSV drug palivizumab (28). While intravenous injection of Ad5-anti-RSV mAb produced high mAb titers at three days post-administration, intrapleural injection of AAVrh.10-anti-RSV mAb took four to eight weeks to reach high mAb titers in the serum, but reduced RSV viral load for at least 21 weeks post-injection.

### Malaria

Malaria presents many unique challenges for vaccine development (278). Malaria is caused by many species of parasites within the genus *Plasmodium*. These parasites, particularly *P. falciparum*, undergo complex life cycles that transmit between humans and mosquitos, and between different host tissue types (279). All of these features increase antigen diversity and pose difficulties for vaccine target selection. For reasons not completely understood, memory B cells against malaria do not persist very long in either natural infections or in vaccination attempts, resulting in the lack of persistent humoral immunity and repeated infections (280–282).

To overcome the inability for humans to retain humoral immunity towards *P. falciparum*, rAAV vectors can be used to mediate long-term expression of the malarial antigens, thus enabling continued stimulation of the immune system. Given that AAVs naturally have low immunogenicity, AdV formulations may be added to improve the immune response raised against the parasite. For example, in a dual-vector strategy, Ad5 and rAAV1 were co-packaged with the *P. falciparum* circumsporozoite protein (PfCSP) and its sexual stage P25 protein (Pfs25). PfCSP is relatively conserved across different *Plasmodium* species, while immunity against Pfs25 is thought to block transmission (283). The Ad5-*Pfs25-PfCSP* vector was given to mice *via* intramuscular injection, followed by AAV1-*Pfs25-PfCSP* vector six weeks later (108). The dual-vectored Ad5-prime/AAV1-boost regimen was highly effective in mice for both full protection and transmission-blocking activity against transgenic *P. berghei* parasites expressing the corresponding *P. falciparum* antigens (108). Remarkably, antibody responses raised in this manner were sustained for over nine months after boosting, and maintained high levels of transmission-reducing activity (108, 109).

Alternatively, rAAV vectors are capable of mediating mAb expression outside of B cells, thus escaping malaria-mediated B cell suppression. To show this ability, rAAV2 vectors expressing human mAbs against PfCSP were delivered to mice *via* intramuscular injection (107). This method offered sterile immunity against the rodent *Plasmodium berghei* strain *via* mosquito bites in all mice that expressed 1 mg/mL or more of the mAb. Expression of mAb also lasted for 8 to 11 weeks, or at the end points of the experiment. It remains to be determined whether rAAV-delivered recombinant mAbs can persist longer than naturally formed anti-malaria mAbs.

### HCV

HCV is a single-stranded, positive-sense RNA virus that uses an error-prone RNA polymerase for genome replication (284). This feature results in high inter-host and intra-host genetic variability that limits the effectiveness of humoral immunity. The non-structural proteins of HCV contain several highly conserved regions with potential to act as vaccine epitopes (285). Intravenous injections of rAAV expressing NS5B in mice elicit strong and durable intrahepatic, NS5B-specific, CD8 T cell activation (111). Additionally, rAAVrh32.33 packaged with the HCV E2 envelope protein can elicit a strong antibody response, while rAAVrh32.33 packaged with HCV NS3 or NS3/4 can elicit both antibody and cellular responses (112, 113). Further studies are needed to assess whether these strategies are effective in protecting against HCV infection in NHP models and human subjects.

### Rabies

Rabies is a severe infection of the CNS that is always nearly fatal. It is caused by zoonotic viruses that belong to the Lyssavirus genus, the most common member being rabies virus (RABV). RABV is responsible for the majority of rabies infections in humans (286). Vaccines and antisera are effective at preventing disease onset following RABV exposure (287). However, in areas where rabies is still endemic, awareness and accessibility to these preventative measures are limited (288). Furthermore, no cure is available once symptoms start to appear. Hence, there is an urgent need to develop prophylactic and treatment regimen against RABV.

Hypothetically, the strong tropism of certain rAAV serotypes toward the nervous system might allow rAAV-mediated delivery of therapeutic mAbs to the RABV-affected tissues. This possibility has not been extensively explored for the treatment of rabies. An attempt to use rAAVs for immunization against RABV was performed in mice (124). In this work, rAAVs containing glycoproteins from various strains of RABV (rAAV-G) were administered *via* intramuscular injection. All rAAV-G treatments induced higher neutralizing antibody titers and cytokine responses than the attenuated RABV vaccine LBNSE-GMCSF. Furthermore, rAAV-G protected mice from intracerebral challenges of RABV for nine months post-administration. It is unclear whether rAAV-G can offer protection against RABV post-exposure.

### Human Papillomavirus (HPV)

Chronic infection by HPV subtypes 16 and 18 are the strongest risk factors for cervical cancer development (289, 290). While vaccines are available to prevent new infections, no cure is present for existing infections. HPV-mediated oncogenesis is driven by the viral proteins E6 and E7. They inhibit tumor suppressor proteins p53 and Rb, leading to the arrest of proliferating cells at the DNA synthesis and growth phases of the cell cycle (291–293). This action increases the risk of genomic instability and malignant transformation of host cells (290). Thus, HPV E6 and E7 are being used as vaccination targets. Intramuscular administration of rAAV1 packaged with the E7 and heat shock protein 70 (hsp70) fusion protein (rAAV1-1618E7hsp70) completely protected mice from challenge by E7-expressing tumor cell lines at 5, 12, and 24 weeks post-treatment (114). In addition, when the rAAV1-E7 vector was given eight to ten days after tumor inoculation, the size of the pre-established tumor was significantly reduced. An alternative strategy is to target the HPV major capsid protein L1. rAAV5 and rAAV9 packaged with HPV16 L1 was administered to rhesus macaques *via* intranasal delivery (115). By using rAAV5-*HPV16-L1* as the prime vector and rAAV9-*HPV16-L1* as the boosting vector, neutralizing antibodies were elicited in four out of six animals and mediated protection for seven months post-immunization. This effect was achieved even in the presence of pre-existing anti-AAV9 antibodies.

## Concluding Remarks and Future Directions

The ability to deliver gene expression cassettes *in vivo* has greatly expanded the toolbox to combat infectious diseases. With rAAV-mediated gene delivery, expression of antigens or therapeutic molecules may be achieved and maintained for long periods. Current studies demonstrated that this approach could prophylactically prevent new infections, with the additional possibility of eliminating existing infections in animal models. However, rAAV-mediated immunotherapy is still in its infancy, with many issues to be solved. Future studies are warranted to address several key questions. First, ADAs toward therapeutic mAbs and Ig derivatives are currently the biggest obstacle to long-term mAb expression from the host, but the underlying mechanisms of ADA development are largely a mystery. Elucidating these mechanisms will be critical to the design of rAAV immunotherapeutics that are capable of overcoming ADAs. Second, pre-existing immunity to the rAAV capsid and the transgene prevents a large portion of the world’s population from gaining access to AAV-vectored immunotherapies. Strategies to evade pre-existing anti-rAAV immunity without compromising anti-pathogen immunity need to be devised. Third, given the fact that rAAV can mediate long-term transgene expression, even when the transgene is no longer desired, regulatable immunotherapies ought to be developed to further ensure patient safety. Fourth, novel capsids should be explored in the context of immunotherapy. And finally, the manufacturing process requires major improvement for deploying rAAV vectors in global distributions efforts.

## Author Contributions

WZ and MM wrote the initial draft of the manuscript. WZ, MM, PT, and GG revised and finalized the manuscript. All authors contributed to the article and approved the submitted version.

## Funding

This work was supported by grants from the University of Massachusetts Medical School (an internal grant) and by the NIH (R01NS076991-01, P01HL131471-02, UG3HL147367-01, R01HL097088, U19 AI149646-01).

## Conflict of Interest

GG is a scientific co-founder of Voyager Therapeutics and Aspa Therapeutics, and holds equity in these companies. GG is an inventor on patents with potential royalties licensed to Voyager Therapeutics, Aspa Therapeutics, and other biopharmaceutical companies.

The remaining authors declare that the research was conducted in the absence of any commercial or financial relationships that could be construed as a potential conflict of interest.
